# Reduction and Resolution of a Hiatal Hernia Using Osteopathic Manipulative Treatment: A Case Report

**DOI:** 10.7759/cureus.26558

**Published:** 2022-07-04

**Authors:** Mikhail Volokitin, Anthony Song, Meredith T Peck, Susan Milani

**Affiliations:** 1 Osteopathic Manipulative Medicine, Touro College of Osteopathic Medicine, Harlem, USA; 2 Osteopathic Manipulative Medicine, Touro College of Osteopathic Medicine, Middletown, USA; 3 Anesthesiology, Maine Medical Center, Portland, USA

**Keywords:** osteopathic manipulative treatment, gerd, paraesophageal hernia, sliding hernia, hiatal hernia

## Abstract

Hiatal hernia is a condition where components of the abdominal cavity, most often a part of the stomach, penetrate through the diaphragm and into the chest cavity. The symptoms of hiatal hernias may differ secondary to their type and severity. The two main types of hiatal hernias are sliding and paraesophageal. Sliding hernias, which are more common and less of a cause for concern, are when the upper portion of the stomach and junction between the stomach and esophagus slides up into the thoracic cavity through a weakened diaphragm. These hernias account for the majority of all hiatal hernias, and their symptoms mimic those of gastroesophageal reflux disorder (GERD) due to laxity in the lower esophageal sphincter. Paraesophageal hernias occur when parts of the stomach and other abdominal organs protrude through the hiatus adjacent to an intact and in-place esophagus and stomach.

Obesity and old age are risk factors for hiatal hernias, but they can occur in patients of any age and gender. Although some hiatal hernias may be asymptomatic, patients with positive symptoms may complain of heartburn, regurgitation of liquids and food back into their mouths, dysphagia, or discomfort and pain in the stomach or esophagus. Hiatal hernias are preferentially diagnosed with proper imaging, mainly with an upper gastrointestinal barium study, or by upper gastrointestinal endoscopy. The treatment for hiatal hernias depends on their severity and surgical repairs, if needed, are mostly done laparoscopically.

In this case of a patient with a 3 cm hiatal hernia diagnosed with the help of esophagogastroduodenoscopy (EGD), the treatment did not require surgery. Instead, osteopathic manipulative treatment (OMT) was used to restore the functionality of the gastrointestinal tract and the placement of the gastroesophageal junction. The patient’s symptoms were found to have improved after the application of OMT alone, with no symptoms of hiatal hernia and resolution of her somatic dysfunctions. The results of this case study suggest that OMT can be effectively utilized to treat the symptoms of hiatal hernias and may also be effective as a curative method as well.

## Introduction

Hiatal hernia describes a condition where there is superior herniation of abdominal contents through the diaphragm [1]. Multiple types and classifications of herniation lead to difficulties in assessing disease prevalence. While many sliding hernias, known as type I, are asymptomatic, patients may experience symptoms of gastroesophageal reflux disorder (GERD) due to laxity in the lower esophageal sphincter. Complications of sliding hiatal hernias are usually related to acid reflux, whereas complications of paraesophageal hernias, type II, can include volvulus, bleeding, and even respiratory compromise [2]. Surgical repair of the hernia is indicated in symptomatic patients to manage complications; however, these surgical interventions can cause more serious complications. In the western population, hiatal hernias are a common occurrence with an estimated prevalence of 15-20% and are far more common in the aging and overweight population [3].

## Case presentation

**Figure 1:**
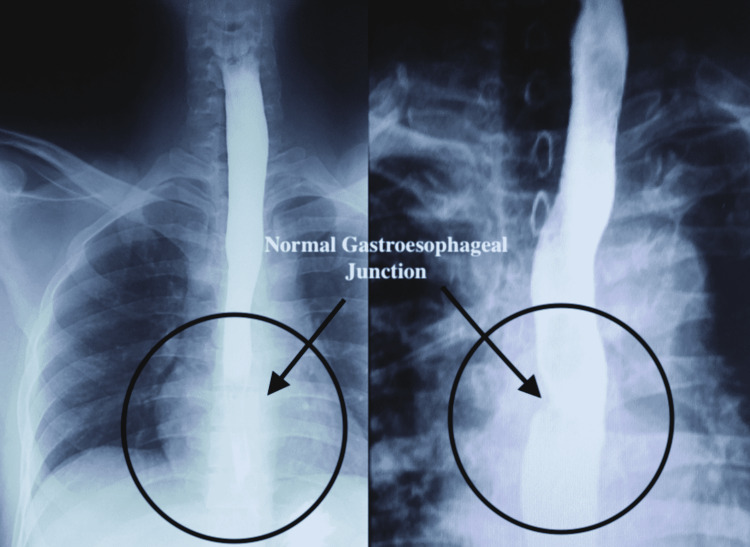
Normal gastroesophageal sphincter on barium swallow with arrows pointing at normal passing of barium through esophagus.

**Figure 2:**
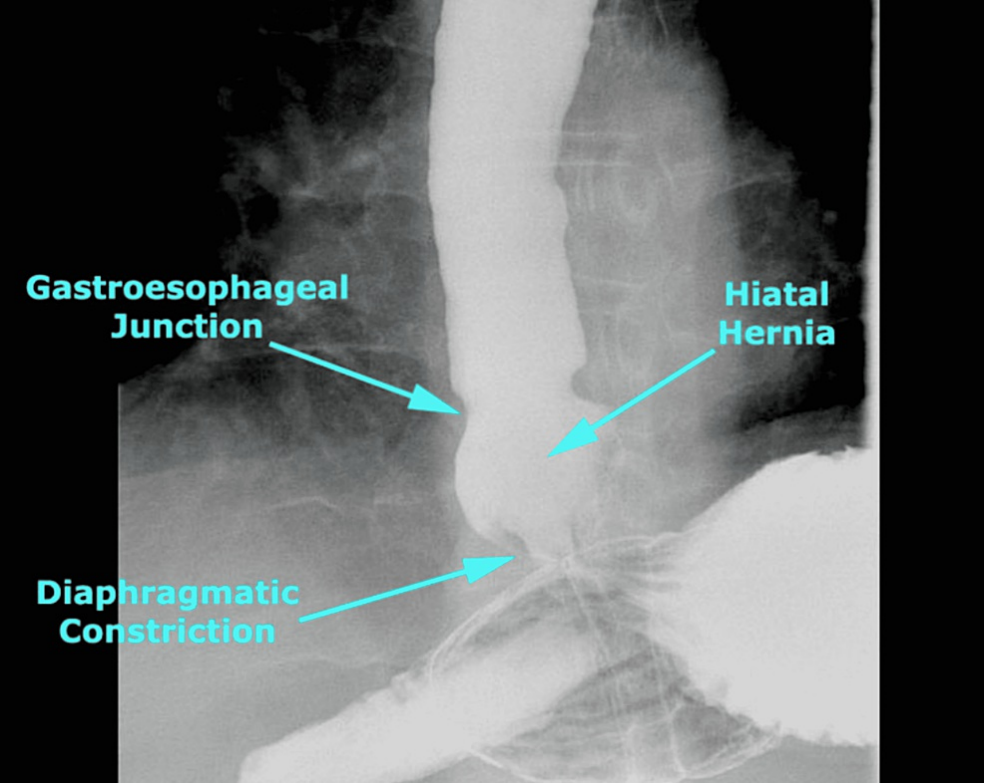
Hiatal hernia on barium swallow.

We present a case of a 71-year-old female complaining of heartburn, nausea, pain, heaviness in the stomach, and nighttime reflux (GERD), lasting on and off for three years. In the past six months, her complaints became more severe and more frequent. She was diagnosed with a 3 cm hiatal hernia by esophagogastroduodenoscopy (EGD) on January 26, 2016. Hiatal hernias can be diagnosed in a number of ways, including EGD and barium swallow. Figure 1 depicts a normal gastroesophageal junction on a barium swallow, while Figure 2 depicts a hiatal hernia on a barium swallow [4,5].

The patient initially tried omeprazole (40 mg once daily) and ranitidine (300 mg once daily) in separate courses for eight weeks each as prescribed by her primary care physician for relief of her symptoms. No proton pump inhibitors (PPIs), calcium carbonate, and herbal or other remedies were used. The patient changed her diet avoiding acidic, caffeinated, and high-fat foods, but after these failed, she sought osteopathic treatment.

On her osteopathic physical exam, the palpatory findings reveal uneven pressure in the abdominal cavity. Her rib cage was rotated to the right with uneven diaphragm tension, with the left hemidiaphragm showing greater restriction than the right. Cervical-thoracic junction of the spine was also flattened. Multiple somatic dysfunctions were diagnosed with the most restriction noted at the thoracic-lumbar junction. Restrictions were also appreciated at the left sternocostal articulation 3-7 with diffuse sternal restrictions mainly at the level of 5-7 bilaterally. Of note, peristalsis of the esophagus was markedly reduced in downward movement. All findings are based on palpatory assessment.

This patient did not have any other past medical or surgical history of significance with no contraindications to receiving osteopathic manipulative treatment (OMT). The following techniques were applied: balanced ligamentous treatment (BLT), myofascial release, muscle energy, and OMT in the cranial field. 

Course of treatment

The patient was treated by a board-certified osteopathic physician with 25 years of osteopathic manipulative medicine (OMM) practice at his private office and 12 years of teaching OMM in an osteopathic college. The patient was treated with OMT using BLT, OMT in the cranial field, muscle energy, and myofascial release to reduce the severity of somatic dysfunctions to restore normal anatomy and functioning to the affected regions. She tolerated the treatment well with no complaints. 

The patient received four osteopathic treatments between the initial diagnosis of a hiatal hernia on January 26, 2016, with all symptoms gradually diminishing. The same treatment modalities were used on each visit. On osteopathic reevaluation on May 5, 2016, esophageal motion had been restored, with no trace of her hiatal hernia. The patient reported complete relief of her reflux symptoms, with no evidence of esophagitis, a hiatal hernia, or mucosal abnormalities at the lower esophageal sphincter. No other complications were seen or reported. She continuously endorsed the improved quality of life with exponential enhancement following each OMT session. There was no follow-up after four visits. She was seen in the office several years later for musculoskeletal complaints resulting from a motor vehicle accident (MVA). The patient presented no GI complaints at that time except occasional mild nausea, which was short in duration and did not require any over-the-counter (OTC) medicine.

Results

After four half-hour OMT sessions, EGD was repeated and showed no evidence of a hiatal hernia. On further visits, the patient did not present any symptoms related to hiatal hernias, and no other somatic dysfunctions were diagnosed. To our knowledge, this is the first case of a hiatal hernia successfully being reduced using OMT as the solitary treatment modality, confirmed by EGD.

## Discussion

Traditionally, the allopathic approach to treating a patient with a hiatal hernia would depend on the severity of their symptoms and the degree of herniation determined via certain diagnostic tests. Patients would most likely present with symptoms like GERD, including heartburn, chest pain, burping, dysphagia, and vomiting [2]. The patient may also experience bloating, belching, a bitter taste in their mouth and the back of their throat, and even shortness of breath. Patients with a past surgical history of hiatal hernia repairs could also experience recurrence of their hiatal hernias, in some cases with a recurrence rate of 20% [6].

With symptoms indicating a hiatal hernia, various diagnostic modalities are available to determine and assess the hernia. Barium swallow involves the patient drinking a chalky liquid containing barium, which can be visualized by a fluoroscopy machine as it passes through the hernia and through the upper GI tract. Endoscopy involves utilizing an endoscope to view the inside of the upper gastrointestinal tract. Other tests include high-resolution manometry, a pH test, and gastric emptying tests [7]. Once properly diagnosed, the hernia is either treated symptomatically, such as with proton-pump inhibitors or other GERD medications, or with surgical repair.

The treatment and workup for a patient with a hiatal hernia through the lens of an osteopathic physician may allow for additional benefits and relief of symptoms to the patient. The osteopathic approach would first consider the functionality of the gastroesophageal junction, which functions as a sphincter for the distal esophagus, relaxing upon swallowing and preventing gastroesophageal reflux during esophageal inactivity. The inability to contract and properly synchronize these contractions indicates a dysfunctional gastroesophageal junction. Physiologic considerations help to explain the occurrence of the hernia. The negative intrathoracic pressure, measured at -5cm H2O, compared to the positive intraabdominal pressure, measured between +5cm to +10cm H2O, creates a force at the gastroesophageal junction, making the stomach susceptible to superior herniation [8].

Neurologic consideration aids in determining which OMT the patient with a hiatal hernia would derive the most benefit from. Increased intrathoracic pressure secondary to the presence of the hernia results in intense vagal nerve stimulation and worsening symptoms of GERD [9]. Anatomically, the cervical-gastric chain of fascia runs from the hyoid to the diaphragm with superficial layers easily accessible to manipulation, allowing access to deep tissues. Restrictions in the attachments to the diaphragm should be evaluated, as hypertonicity and dysfunction of these structures may worsen the herniation. These structures include the ribs, sternum, thoracolumbar junction, and lumbar vertebrae. Application of BLT, osteopathic cranial manipulative medicine, muscle energy, and myofascial release allows for the inhibition of an overly stimulated vagus nerve and correction of somatic dysfunctions around the diaphragm. The vagus nerve carries mainly parasympathetic fibers with both sensory and motor functions [10]. The amplitude of contraction is determined by a balance between intrinsic excitatory cholinergic, inhibitory nitrergic, as well as post inhibition rebound excitatory output to the musculature. This is strongly influenced by vagal efferent motor neurons and this, in turn, is influenced by vagal afferent neurons that send bolus information to the solitary nucleus where programmed activation of the vagal motor neurons to the smooth muscle esophagus is initiated. This indicates that a low-amplitude esophageal contraction can be caused by a multitude of factors and, therefore, many pathways can be potentially explored to restore normal esophageal peristalsis [11].

As cervical region tightness is hypothesized to cause intestinal problems through the vagus nerve, relaxation of the hypertonic tissues of the cervical region may be a promising treatment for abdominal discomfort and symptoms of GERD. Myofascial release is a treatment technique that engages continual palpatory feedback to achieve the release of myofascial tissues. BLT was used to address ligamentous articular strains. Muscle energy treatment was applied to resolve a Golgi tendon reflex maintaining corresponding somatic dysfunction [12].

## Conclusions

Successful osteopathic treatment requires a thorough understanding of the anatomy and physiology of the gastroesophageal junction and its associated structures. Considerations must be made as to the fascial, muscular, bony, and soft tissue structures and their relatedness, as well as innervation and the striking pressure differential between the thoracic and abdominal cavities. Utilization of OMT was shown in this patient to not only completely alleviate symptomatology but to eliminate the patient’s need for surgical intervention and the possibility of related complications therein. This case suggests there is a role for OMT not only in the treatment of symptoms associated with hiatal hernias, namely symptoms of GERD, but OMT may also serve as the pathologic treatment. To our knowledge, this is the first case of a hiatal hernia, confirmed by EGD, to be successfully reduced with the resolution of symptoms using OMT.

The results indicate that OMT can be useful to restore the patient’s health and well-being to a baseline level prior to the onset of symptoms while decreasing the need for surgery. As this is a case study involving one patient, larger studies may be needed to provide statistically significant evidence for the effectiveness of OMT in treating hiatal hernias.
